# Advancing Community-Based Falls Prevention Programs for Older Adults—The Work of the Administration for Community Living/Administration on Aging

**DOI:** 10.3389/fpubh.2017.00004

**Published:** 2017-02-03

**Authors:** Kristie Kulinski, Casey DiCocco, Shannon Skowronski, Phantane Sprowls

**Affiliations:** ^1^Administration for Community Living, Washington, DC, USA

**Keywords:** falls prevention, falls, community-based programs, evidence-based programs, Federal Government

## Abstract

The mission of the Administration for Community Living (ACL) is to maximize the independence, well-being, and health of older adults, people with disabilities across the lifespan, and their families and caregivers. In direct alignment with this mission is ACL’s support of evidence-based falls prevention programs in communities throughout the United States. Since 2014, the Administration on Aging (AoA), part of ACL, has invested nearly $14 million in entities such as state agencies, nonprofits, and universities to expand access to proven community-based falls prevention programs. The initiatives supported by ACL/AoA bring to bear two primary goals—(1) to significantly increase the number of older adults and older adults with disabilities at risk for falls who participate in evidence-based community programs to reduce falls and falls risks; and (2) to implement innovative funding arrangements, including contracts, partnerships, and collaborations with one or more sustainability partners to support these programs during and beyond the grant period. Support from ACL/AoA has significantly increased the availability of evidence-based falls prevention programs in funded communities, as well as enhanced the network’s sustainable delivery infrastructure to promote continued access to these critical programs beyond the scope of grant funding. This article highlights the successful rollout of ACL/AoA’s falls prevention initiative.

## Introduction

The Administration for Community Living (ACL) was created in 2012 as a new agency under the U.S. Department of Health and Human services, bringing together previously separate federal offices and agencies administering programs to benefit older adults, people with disabilities, and caregivers. We now know that these programs are stronger together, and today, ACL works every day to pursue our mission to maximize the independence, well-being, and health of older adults, people with disabilities across the lifespan, and their families and caregivers. A guiding principle that ties all ACL programs together is that everyone has a right to live and contribute in their communities.

Falls and their consequences are one of the biggest risks to the health and independence of an older adult in the United States. Falls can have a significant impact on a wide variety of health factors, they can be deadly for many older adults, and they often result in high costs for the individual and the health-care system as a whole.

One in four Americans aged 65 and older falls every year (1). More than 95% of hip fractures are caused by a fall, and falls are also the most common cause of traumatic brain injuries (2, 3). Recent data show that each year 2.8 million older people are treated in emergency departments as a result of falls, and over 800,000 patients are hospitalized (4). Adjusted for inflation, the direct medical costs for fall injuries are $31 billion annually (5). Falls have also been noted as the leading cause of injury death among older adults (6).

Fortunately, research has shown that falls and falls risks can be reduced through systematic risk identification and targeted intervention, including a combination of clinical and community-based interventions (7). Multiple evidence-based community programs have been shown to reduce falls and/or falls risk factors (8–10) as well as to provide a positive return on investment (11).

Within ACL, the Administration on Aging (AoA) has a long history of supporting disease prevention and health promotion efforts. For more than a decade, AoA discretionary grants have helped build an infrastructure to increase access to and sustain evidence-based disease prevention and health promotion programs. Falls prevention is a key pillar of our agency’s work in this area. Through proven programs offered throughout the country, older adults and people with disabilities have the opportunity to mitigate their falls risk and participate in their communities to a greater extent.

## Evidence-Based Falls Prevention Programs

The risk factors associated with older adult falls are numerous and varied. But, whether physiological, pharmacological, behavioral, or environmental, these risk factors are also largely modifiable. Over the past three decades, health researchers have been developing and studying interventions that can modify these risk factors and keep older adults from falling.

Research from fields as varied as occupational therapy, behavioral health, and industrial design has resulted in a sophisticated and expanding knowledge of how and why older adults fall, and ways to reduce their likelihood to fall. This landmark special issue showcases this growing literature base.

Yet, despite our increasing understanding of falls and falls risk, the rate of older adult falls continues to rise (12). Even when adjusted for our nation’s increasing older adult population, the proportion of those older individuals who are falling is going up (12). These numbers demonstrate the need to find ways to bring the established scientific understanding of falls and falls prevention to people’s homes, communities, and health-care institutions. Luckily, there are evidence-based programs that do just that.

Evidence-based programs in the field of health promotion, broadly, are an established set or sequence of activities and inputs, delivered in a prescribed way, designed to result in specific outcomes. In other words, these are programs that can be implemented in the same way across different locations and times, and the participants should show similar outcomes. Such programs have been studied in controlled settings, and the evidence that forms the basis of these programs’ effectiveness is collected through formalized methods of data collection, with appropriate prioritization and analyses of the results.

As the health and aging services communities have increasingly recognized the tremendous burden of older adult falls, clinical and public health researchers have developed evidence-based programs that are proven to reduce falls and falls risk. These programs have been studied with older adults living in community settings and have been shown to result in positive outcomes for the participants. These programs are being implemented in community and clinical settings across the country with older adults of diverse backgrounds, abilities, and languages.

## ACL’s Investment in Evidence-Based Falls Prevention Programs

Administration for Community Living funds the dissemination and implementation of evidence-based programs for older adults through a number of avenues. Over the past decade, a variety of resources have propelled widespread adoption of evidence-based community falls prevention programs throughout the country. In 2003, the AoA [in collaboration with the Centers for Disease Control and Prevention (CDC) and other partners] supported the widespread efforts of states, communities, and researchers to translate evidence-based health and prevention programs into community settings and develop tools to promote replication of these programs. From 2006 through 2011, AoA awarded grants to 24 states to develop infrastructure and partnerships to work toward embedding these proven programs within communities. In addition to supporting evidence-based falls management programs, these grants supported Stanford University’s Chronic Disease Self-Management Program, physical activity, depression, and behavioral change programs.

Between 2006 and 2011, more than 31,000 older adults in 18 states were reached *via* AoA-supported falls management programs. These programs were offered at more than 1,500 community-based sites, such as senior centers, senior housing facilities, faith-based organizations, health-care organizations, and other entities (13).

In 2014, for the first time, ACL received dedicated funding through the Affordable Care Act’s Prevention and Public Health Fund (PPHF) to support evidence-based community falls prevention programs. The purpose of this funding is twofold: (1) to build upon the delivery and distribution systems that have been developed for evidence-based falls prevention community programs across the nation and (2) to leverage national, state, and local falls prevention efforts that align with these efforts, such as the work of CDC’s Injury Prevention Center and the National Council on Aging’s Falls Free^©^ Initiative.

Between 2014 and 2016, ACL funded 32 grantees to support the implementation of evidence-based falls prevention community programs (14). The goals of these grants are to significantly increase the number of older adults and older adults with disabilities, at risk for falls, who participate in evidence-based community falls prevention programs. Concurrent goals are increasing the sustainability of these programs through innovative funding arrangements and embedding the programs into the nation’s health and long-term services and supports systems. Grant recipients include non-profit organizations, universities, and state, local, and tribal governments. In 2014 and 2016, a special emphasis was placed on reaching American Indian, Alaskan Native, and Native Hawaiian elders, populations for whom falls are the leading cause of unintentional injury deaths (15).

Beginning in 2014, ACL also funded its first-ever National Falls Prevention Resource Center, housed at the National Council on Aging (16). This Center works to increase public education about the risks of falls and how to prevent them, as well as to support and stimulate the implementation and dissemination of evidence-based community programs and strategies that have been proven to reduce the incidence of falls among seniors. The Center has produced a variety of resources and tools to help increase falls prevention education, as well as to support the dissemination and sustainability of falls prevention programs.

Through careful planning and strategic partnerships, ACL’s PPHF Falls Prevention grantees have been successful in substantially increasing the reach of proven falls prevention programs. To date, our 2014 and 2015 grantee cohorts have reached over 26,000 older adults with evidence-based community programs—with the 2014 grantees exceeding their target numbers for persons reached (124% of their cumulative 2-year goal). Programs being implemented include:

- A Matter of Balance: this is a structured, class-based program that helps older adults overcome their fear of falling and increase their activity levels. The class content addresses a number of falls risk factors including environment, balance, and physical activity (10).
- Otago Exercise Program: this program is a series of 17 strength and balance exercises delivered by a physical therapist in the home. The physical therapist assesses, coaches, and progresses patients over the course of 6 months to 1 year (17).
- Stay Safe, Stay Active: this program consists of weekly structured group sessions of moderate-intensity exercise, held in community settings, with additional exercises performed at home (18).
- Stepping On: this program teaches participants to recognize falls risks in their physiology behaviors and environments, as well as exercises and activities to reduce these risks (9).
- Tai Chi for Arthritis: this therapeutic movement-based intervention helps people with arthritis improve their strength, flexibility, balance, and stamina, in order to help prevent falls (19).
- Tai Ji Quan: Moving for Better Balance: this program is delivered in two 1-h sessions each week for 24 weeks. Each session consists of warm-up exercises; core practices, which include a mix of practice of forms, variations of forms, and mini-therapeutic movements; and brief cool-down exercises (20).

Workshops were hosted by and took place in a variety of locations—the most common of which were senior centers (24%), residential facilities (19%), health-care organizations (14%), and faith-based organizations (9%). Programs were offered in a variety of languages, including Chinese, Vietnamese, Spanish, Navajo, Hmong, Korean, and Cambodian. Grantees have also developed strategies and mechanisms to help reach persons with disabilities, such as partnering with Centers for Independent Living and developing resources to ensure that persons with low vision and/or hearing are able to fully participate in falls prevention programs (21). Our data also tell us that grantees are successfully reaching an older (average age of 76), vulnerable population who has significant risk factors for falls. A history of previous falls is a significant predictor of future falls, and nearly two-thirds of program participations report having fallen at least once in the last 3 months. Additionally, nearly 40% of participants reported limited physical activity, which can also increase an older adult’s risk of falling (Table 1).

**Table 1:** **Characteristics and health status of participants reached by falls prevention programs (2014–2015)**.

	A Matter of Balance (*n* = 17,615)	Stepping On (*n* = 3,345)	Tai Ji Quan (*n* = 3,987)	Tai Chi for Arthritis (1,229)	FallScape (*n* = 174)	Stay Safe, Stay Active (144)	Otago (*n* = 71)	All programs (*n* = 26,565)
**Demographics**	**%**	**%**	**%**	**%**	**%**	**%**	**%**	**%**
Female	80.9	76.8	81.0	81.2	68.6	84.6	80.3	80.3
Age, M (SD)	76.8 (12.4)	76.8 (8.3)	73.3 (9.2)	72.1 (8.7)	76.9 (10.4)	67.5 (10.4)	75.9 (9.5)	76.1 (11.5)
Race/ethnicity								
White	66.8	80.7	56.8	54.7	96.0	68.1	60.6	66.6
Black/African-American	7.0	<1	2.4	15.3	0.0	1.4	35.2	5.9
Asian-American	1.9	<1	4.8	2.8	0.0	0.0	1.4	2.2
Hispanic/Latino	4.5	2.2	1.4	3.7	1.1	<1	2.8	3.6
Hawaiian/PI	<1	<1	<1	<1	0.0	0.0	0.0	<1
American Indian/Alaska native	<1	1.9	<1	6.2	<1	20.1	0.0	1.1
Multi-racial	<1	<1	<1	<1	<1	9.0	0.0	<1
Unknown	18.5	13.4	33.5	16.3	1.7	<1	0.0	19.8
Education								
Some high school	10.4	4.4	9.0	17.4	18.8	2.9	28.6	9.9
High school graduate/GED	25.6	22.4	16.0	19.3	37.0	29.2	40.0	23.9
Some college/vocational training	30.9	33.7	27.7	24.1	16.9	32.8	25.7	30.4
College graduate or higher	33.0	39.6	47.3	39.2	27.3	35.0	5.7	35.8
Live alone	51.3	46.2	40.4	37.1	41.9	26.8	52.9	39.4
**Health status**								
3 or more chronic conditions	16.3	18.5	8.1	17.4	47.1	22.9	49.3	15.7
Limited activity	39.0	49.3	31.6	29.8	56.0	29.6	55.1	39.4
Self-rated health								
Excellent	5.9	5.6	5.6	5.8	6.1	4.2	4.9	5.8
Very good	30.1	28.6	29	29.9	35.7	34.7	22	29.8
Good	46.5	47.5	47.3	45.1	46.9	40.3	51.2	46.7
Fair	15.8	16.5	16.9	17.1	11.2	19.4	17.1	16.1
Poor	1.6	1.8	1.1	2.1	0	1.4	4.9	1.6
No. of falls in last 3 months								
None	67.6	62.8	77.2	77.8	39	80.9	57.4	68.4
1	17.8	20.1	13.8	13.2	27.9	11.8	18.5	17.4
2 to 3	11.2	13.5	7.2	7.1	24.4	5.9	18.5	11
4 or more	3.4	3.6	1.8	1.8	8.7	1.5	5.6	3.2

Grantees have made great strides in strengthening the infrastructure and delivery systems necessary not only to reach participants but also to embed and sustain these programs to enroll new participants after the point at which their limited grant funding ends. A few examples of broad strategic approaches for advancing the sustainability of these programs include:

- The use of a “Hub,” i.e., a centralized entity that includes multiple partner organizations and provides training, technical assistance, quality assurance, and administrative support for falls prevention programs, as well as a menu of other evidence-based programs. Grantees have found that a Hub approach has several advantages, including the ability to centralize operations, leverage resources, and encourage more efficient contracting to promote program sustainability.
- Expanding partnerships with health-care organizations (i.e., hospitals, Federally Qualified Health Centers, insurers, etc.) to build in referral to or embed community falls prevention program within these organizations, as well secure payment for the programs.
- Embedding program delivery into existing funding streams (i.e., Title IIID of the Older Americans Act, CDC Injury Prevention, state and local government, employee/retiree benefit programs, etc.).

Specific examples of grantee sustainability approaches include:

- The Colorado Department of Public Health and Environment and New York State Department of Health have partnered with Level 1 Trauma Centers to embed programs such as Stepping On and Tai Chi: Moving for Better Balance.
- Florida Health Networks has developed a statewide infrastructure capable of offering evidence-based falls prevention programs with reimbursement from managed care organizations.
- The Healthy Living Center of Excellence in Massachusetts has established statewide capacity through contracted relationships with more than 80 diverse organizations and is integrating evidence-based falls prevention programs in medical homes, accountable care organizations, and other shared risk pilots.

## Conclusion

The ACL remains steadfast in our commitment to help older adults prevent falls. Supporting falls prevention programs is in direct alignment with our agency’s mission, as well as our commitment to the fundamental principle that older adults and people with disabilities should be able to live where they choose, with the people they choose, and participate fully in their communities. While falls are not an inevitable part of aging, they can certainly trigger dire consequences for older adults. Knowing that a fall can result in decreased independence and impact the ability to actively engage in preferred activities or even remain safely in one’s home, it is imperative that we equip older adults with the necessary skills and tools to prevent a fall from happening in the first place.

We are proud of what our diverse network of federal, state, local, and tribal partners throughout the country has accomplished over the past decade. Tens of thousands of older adults have benefitted from evidence-based falls prevention programs, and that number is growing each and every day. We are also mindful of the challenges and opportunities that lie ahead. With roughly 10,000 people turning 65 every day, it is imperative that as a collective network we identify and seize various opportunities to scale and sustain these impactful interventions (22). For example, potential for community/clinical linkages exist within innovative health-care delivery and financing models such as Accountable Care Organizations and Patient-Centered Medical Homes. These models present a unique opportunity for community-based organizations to demonstrate the value of proven falls prevention programs as it relates to both improved health and cost savings (23). Only through collaboration and the leveraging of diverse, though often scarce, resources, will we realize the profound impact on falls prevention that is necessary to make an impact at a population level, and ACL is excited to be a key player in these efforts.

## Author Contributions

KK, CD, SS, and PS each wrote and edited portions of the manuscript.

## Conflict of Interest Statement

The authors declare that the research was conducted in the absence of any commercial or financial relationships that could be construed as a potential conflict of interest.
